# Impact of attending neonatologist presence on neonatal intubation success and adverse events: a cohort study

**DOI:** 10.1038/s41372-025-02551-3

**Published:** 2026-01-27

**Authors:** Clement Trinh, Kate A. Hodgson, Marnie Downes, Brett J. Manley, Marta Thio, Michael-Andrew Assad, Katharina Bibl, Shilpi Chabra, Cassandra DeMartino, Stephen DeMeo, Kristen Glass, Heidi Herrick, Bin Huey Quek, Sabine Iben, Philipp Jung, Jae Kim, Ayman Abou Mehrem, Ahmed Moussa, Michael Narvey, Joyce O’Shea, Nicole Pouppirt, Mihai Puia-Dumitrescu, Jennifer Rumpel, Rebecca Shay, David Tingay, Michelle Tyler, Jennifer Unrau, Michael Wagner, Paul Wildenhain, Akira Nishisaki, Elizabeth E. Foglia

**Affiliations:** 1 Newborn Research Centre, The Royal Women’s Hospital, Melbourne, VIC Australia; 2 Murdoch Children’s Research Institute, Parkville, VIC Australia; 3 Department of Obstetrics, Gynaecology and Newborn Health, The University of Melbourne, Melbourne, VIC Australia; 4 Division of Neonatology, CHU Sainte-Justine, Montreal, QC Canada; 5 Medical University of Vienna, Division of Neonatology, Pediatric Intensive Care and Neuropediatrics, Comprehensive Center of Paediatrics, Vienna, Austria; 6 Division of Neonatology, Department of Pediatrics, University of Washington, and Division of Neonatology, University of Washington and Seattle Children’s Hospital, Seattle, WA USA; 7 Department of Pediatrics, Yale University, New Haven, CT USA; 8 Division of Neonatology, WakeMed Health and Hospitals, Raleigh, NC USA; 9 Division of Neonatal-Perinatal Medicine Department of Pediatrics Penn State Milton S. Hershey Medical Center Penn State College of Medicine, Seattle, WA USA; 10 Department of Pediatrics/Division of Neonatology at the Children’s Hospital of Philadelphia, Philadelphia, PA USA; 11 Department of Neonatology, KK Women’s and Children’s Hospital, Singapore, Singapore; 12 Department of Neonatology Cleveland Clinic Children’s, Cleveland, OH USA; 13 University Hospital Schleswig-Holstein, Campus Luebeck, Pediatric Department, Luebeck, Germany; 14 Perinatal Institute, Department of Pediatrics, Cincinnati Children’s Hospital Medical Center, University of Cincinnati College of Medicine, Cincinnati, OH USA; 15 Department of Pediatrics, University of Calgary, Calgary, AB Canada; 16 Neonatal-Perinatal Medicine University of Manitoba, Winnipeg, MB Canada; 17 Royal Hospital for Children, Glasgow, UK; 18 Department of Pediatrics/Division of Neonatology, Ann & Robert H. Lurie Children’s Hospital of Chicago, Chicago, IL USA; 19 Department of Pediatrics/Division of Neonatology, University of Washington and Seattle Children’s Hospital, Seattle, WA USA; 20 Department of Pediatrics, University of Arkansas for Medical Sciences, Little Rock, AR USA; 21 Department of Pediatrics, Division of Neonatology, University of Colorado School of Medicine, Aurora, CO USA; 22 Neonatal Research Murdoch Children’s Research Institute Melbourne, Australia, Department of Paediatrics, University of Melbourne, Neonatology Royal Children’s Hospital, Melbourne, VIC Australia; 23 Division of Neonatology, Children’s Hospital at Dartmouth-Hitchcock Medical Center, Lebanon, NH USA; 24 Section of Newborn Critical Care, University of Calgary, Calgary, AB Canada; 25 Division of Neonatology, Pediatric Intensive Care and Neuropediatrics, Department of Pediatrics, Comprehensive Center for Pediatrics, Medical University of Vienna, Vienna, Austria; 26 Division of Neonatology, Children’s Hospital of Philadelphia, Philadelphia, PA USA; 27 Department of Anesthesiology, Critical Care, and Pediatrics, University of Pennsylvania Perelman School of Medicine, Philadelphia, PA USA; 28 Children’s Hospital of Philadelphia—KOPH, Philadelphia, PA USA; 29 Cincinnati Hospital Medical Center, Cincinnati, OH USA; 30 Dartmouth Hitchcock Medical Center, Lebanon, NH USA; 31 The University of Washington Medical Center (UWMC), Seattle, WA USA; 32 University of Wisconsin—American Family Children’s Hospital/Unity Point Meriter, Madison, WI USA; 33 Yale-New Haven, New Haven, CT USA

**Keywords:** Health care, Paediatrics

## Abstract

**Objective:**

To evaluate the effect of attending neonatologist presence on first attempt neonatal intubation success and adverse events.

**Study design:**

Retrospective review of National Emergency Airway Registry for Neonates (NEAR4NEOS) intubations October 2014–December 2022. Univariate and multivariate analyses were performed to estimate associations between attending presence and outcomes.

**Results:**

Among 12,652 intubation encounters, attendings were present for 8391 (66%) intubations by more junior operators. On univariate analysis, attending presence was associated with higher first attempt intubation success (OR 1.11, 95% CI 1.04–1.2). However, on multivariate analysis, attending presence was associated with lower first attempt success (aOR 0.78, 95% CI 0.70–0.86) and intubation requiring ≥3 intubation attempts (aOR 1.39, 95% CI 1.21–1.60).

**Conclusion:**

After adjustment, attending presence was associated with lower odds of first attempt intubation success. Reasons for this may include appropriate anticipation of high-risk intubations, altered team dynamics or unmeasured confounding biases.

## Introduction

Neonatal endotracheal intubation is an important procedure required for the sickest infants. With advances in neonatal intensive care, fewer infants require intubation and therefore there are fewer opportunities for neonatal clinicians to gain proficiency [1]. In adult emergency and critical care settings, first attempt endotracheal intubation success rates are approximately 80% [2, 3]. In contrast, first attempt success rates for neonatal intubation are lower, around 50% overall. Experience of the operator [4, 5], premedication use [6, 7], nasal high flow therapy [8], and video laryngoscope use in inexperienced operators [9] are factors associated with increased success.

Severe desaturation and adverse events are common during neonatal intubation, and occur more often with repeated intubation events [10–12]. The neonatal brain is particularly susceptible to physiological instability associated with attempts at intubation [13, 14]. Thus, maximizing the chances of first attempt intubation success and maintaining patient stability during the procedure are of critical importance.

Greater operator experience increases first attempt intubation success and reduces adverse tracheal intubation-associated events (TIAEs) [15–17]. Having an attending neonatologist present may provide immediate expertise, leadership, and oversight that could increase first attempt success and reduce adverse events. However, the impact of an attending neonatologist supervisor on these outcomes is unknown. The objectives of this study were to explore the factors associated with attending neonatologist presence at intubation, and to estimate the association between attending presence and first attempt intubation success in trainees and TIAEs. The hypothesis was that attending presence would be associated with an increased likelihood of first attempt intubation success, and a decreased likelihood of TIAEs.

## Methods

This study was a retrospective cohort study of prospectively collected data from 19 neonatal intensive care units (NICUs), across the USA, Canada, Australia, Germany and Austria, that contribute data to the National Emergency Airway Registry for Neonates (NEAR4NEOS), an international multicenter data registry of neonatal tracheal intubations. Each contributing site has individual approval or exemption to send data to the registry, and the Children’s Hospital of Philadelphia (CHOP) Institutional Review Board granted ethical approval for observational analyses of the database. All methods were performed in accordance with the relevant guidelines and regulations. There was a waiver of parental consent for the use of patient data as a quality improvement activity.

### Data collection

A standardized data collection form was used by each participating NICU to collect patient, provider, practice, and outcome data for all neonatal tracheal intubations. To maintain high levels of data integrity, every NICU developed and adhered to a specific compliance plan to ensure the accurate capture and entry of >90% of intubations. Data from each site were entered into the secured, password-protected Research Electronic Data Capture (REDCap) system [18] hosted by the data coordinating center at CHOP.

Patient-related data captured included gestational age, age in days, and weight at birth and at the time of intubation, co-morbidities, premedication use, location of intubation, and indication for intubation. Provider-related data included the operator’s role and years of experience, and the attending neonatologist's presence at the intubation. Attending neonatologist is the term used for any practicing medical practitioner with specialist qualifications in neonatal critical care, and may be referred to using different terms (for example, consultant) in different regions.

Practice data included the intubation device used, primary intubation or endotracheal tube exchange, and whether premedications were given. Outcome data included whether the intubation was successful, the number of attempts required, and whether there were any TIAEs. An attempt was defined as an airway maneuver that starts with the insertion of the device (i.e., laryngoscope) into the patient’s mouth and ends when the device is removed. First-attempt success was defined as successful intubation on the first attempt by the first airway provider. The NEAR4NEOS registry did not collect data regarding 24-h on-site attending presence or the reasons for an attending being present at an intubation attempt.

### Inclusion and exclusion criteria

All intubations that occurred between October 1st, 2014 and December 31st, 2022 were eligible for inclusion. Intubations where an attending was the initial intubation operator were excluded from the analysis. Intubations that had conflicting or missing data for the primary outcome (first attempt success) or the exposure of interest (attending presence) were also excluded. Following the development of a Directed Acyclic Graph [19] to identify confounding variables to include in statistical modeling, intubations with missing data for these covariates were also excluded.

### Study outcomes

The primary outcome was first attempt intubation success, defined as successful intubation on the first attempt by the first operator.

The secondary outcomes were severe and non-severe tracheal intubation adverse events (TIAEs), as defined by previously published operational descriptions [4], severe oxygen desaturation (defined as ≥20% absolute decrease in peripheral oxygen saturation (SpO_2_) from the highest SpO_2_ documented immediately before the first intubation attempt), and multiple attempts (defined as ≥3 attempts, given the previously demonstrated increased risk of TIAEs and severe oxygen desaturation at this threshold) at intubation [12].

Non-severe TIAEs included esophageal intubation with immediate recognition, mainstem intubation, lip trauma, pain or agitation requiring additional sedation delaying intubation, epistaxis, emesis without aspiration, and dysrhythmia, including bradycardia with heart rate <60 beats per minute. Severe TIAEs included direct airway injury, esophageal intubation with delayed recognition (defined as placement of the endotracheal tube into the esophagus or hypopharynx with a lapse of time and clinical deterioration, such as severe desaturation, before the misplaced tube was removed), emesis with aspiration, laryngospasm, pneumothorax, gum or dental trauma, hypotension requiring intervention, need for cardiac compressions and cardiac arrest.

### Directed acyclic graph (DAG) formulation and covariate selection

**Fig. 1:**
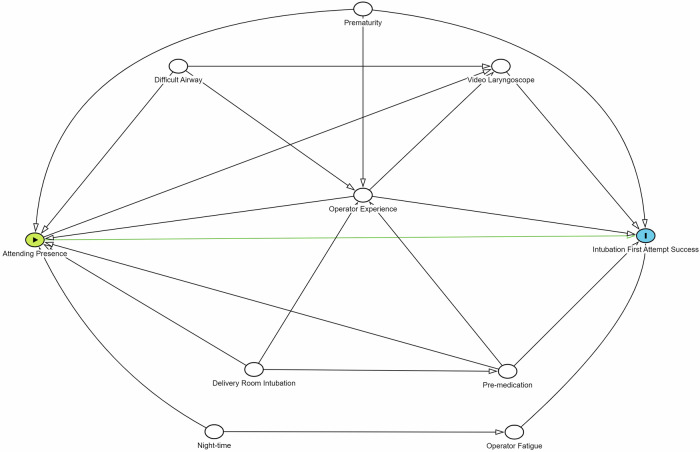
Directed acyclic graph (DAG) for first attempt intubation success depicting assumed causal relationships between variables. In a DAG, a confounder is depicted as a variable that has direct pathways to both the exposure of interest and the outcome.

A directed acyclic graph (DAG) was developed to identify confounders of the hypothesized causal relationship between attending presence and first attempt intubation success (Fig. 1). A DAG represents known, theorized and assumed causal relationships between variables, including the exposure, outcome and confounders [19].

A minimally sufficient adjustment set was identified to establish the smallest set of variables that, when controlled for, adequately adjusted for confounding, thus allowing for an unbiased estimate of the causal effect of the exposure on the outcome [20, 21]. The minimally sufficient adjustment set identified comprised the following confounders: prematurity (<37 weeks’ gestation) [22], a known history of difficult airway (as identified by the operator prior to intubation) [10], operator experience [4, 23], pre-medication prior to intubation [7], use of video laryngoscopy [9], and night-time intubations [24]. The operator’s years of experience were used as an indirect measure of neonatal intubation experience, as physician training level, measured in postgraduate years, has previously been demonstrated to be associated with first attempt intubation success [16]. Video laryngoscope use has been demonstrated to be associated with higher rates of first attempt intubation success, particularly in inexperienced operators.

### Statistical analysis

Summary descriptive statistics were used to describe the patient demographics, using median and interquartile range (IQR) for continuous variables, and using frequencies and percentages for categorical variables. Associations between attending neonatologist presence and patient, provider, and practice characteristics were assessed using Chi-square tests for categorical variables and Kruskal–Wallis tests for continuous variables.

Univariate logistic regression modeling was performed to estimate the unadjusted association between attending presence and each individual primary and secondary outcome, with estimates presented as odds ratios (OR) with 95% confidence intervals (CI). Multivariable modeling using generalized estimating equations with covariate selection guided by the DAG and site clustering accounted for by use of an exchangeable correlation structure was then performed to estimate the adjusted causal effect of attending presence on each outcome, presented as adjusted odds ratios (aORs) with 95% CIs. During the statistical analysis, it was found that there was insufficient power to model the effect of consultant presence on individual TIAEs. The decision was made to present the results of multivariate modeling for composites of adverse outcomes, specifically any non-severe and any severe TIAEs, whilst providing descriptive statistics for individual events.

Correlation between intubations occurring within the same institution due to variations in institution practices or patient demographics was also accounted for in the statistical analysis.

Pre-specified subgroup analyses for the primary outcome were performed with the inclusion in the multivariable model of an interaction term between attending presence and pre-specified subgroups separately: level of experience of the operator (0–2 years, 3–5 years, >6 years), gestational age (<28 weeks’ completed gestation, >28 weeks but <34 weeks’ completed gestation, >34 weeks’ completed gestation), known history of difficult airway, and location of intubation (delivery room or NICU). Statistical analysis was performed using the R programming language (version 4.3.2) [25].

## Results

**Fig. 2:**
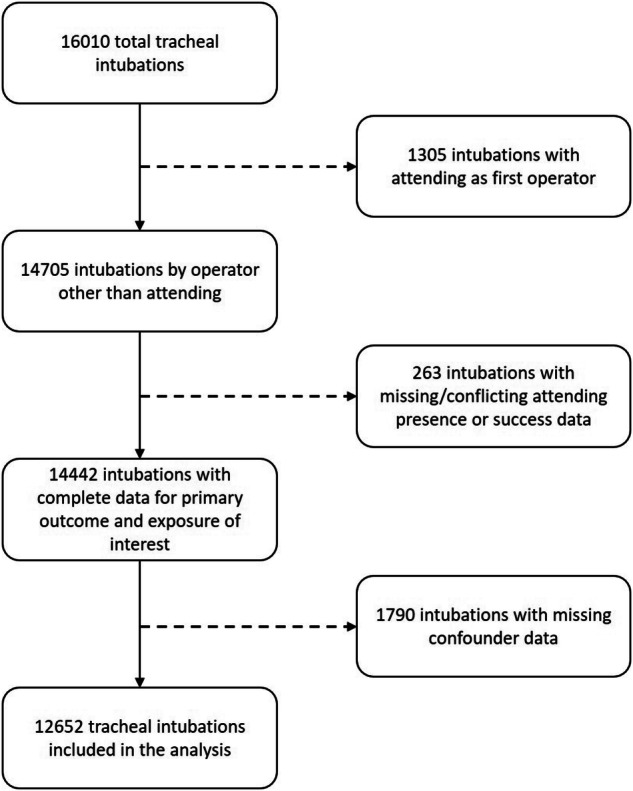
Study flow chart.

Of 16,010 tracheal intubations during the study period, 12,652 were included in the analysis (Fig. 2). A total of 1305 intubations where an attending was the operator were excluded. Of the 1790 intubations that were excluded for missing data for identified confounder variables, 1145 were intubations performed by respiratory therapists or ‘other’ operators for which there were no data for years of experience recorded.

Of the 12,652 included intubations, 8391 (66.3%) had an attending present. Patient, provider and practice characteristic data are summarized in Table 1. Intubation encounters with an attending neonatologist supervisor present were more likely to involve infants who were of greater weight and age, and who had a known history of difficult airway. Intubations with an attending neonatologist present were also more likely to use a video laryngoscope and occur in the delivery room and during the day. The reason for the attending neonatologist being present, and the time at which they attended during the intubation episode, were not recorded.

**Table 1:** Patient, provider and practice characteristics stratified by attending neonatologist presence.

Characteristics (*N* = 12,642)	Attending neonatologist present (*n* = 8383)	Attending neonatologist absent (*n* = 4259)	*p* value
Gestational age at birth—weeks, median (IQR)	29 (25–36)	29 (26–34)	0.28
Age at intubation—days, median (IQR)	3 (0–35)	1 (0–20)	<0.001
Weight at intubation—grams, median (IQR)	1900 (970–3000)	1499 (930–2600)	<0.001
Known history of difficult airway (*n*, %)	635 (7.6)	221 (5.2)	<0.001
Video laryngoscope used^a^ (*n*, %)	3517 (42.0)	760 (17.8)	<0.001
Received pre-medication (*n*, %)^b^	5136 (61.2)	2296 (53.9)	<0.001
Experience level of first operator (*n*, %)			<0.001
0–2 years	2394 (28.6)	1432 (33.6)	
3–5 years	4732 (56.4)	2159 (50.7)	
6+ years	1257 (15.0)	668 (15.7)	
Location of intubation (*n*, %)			<0.001
Delivery room	2316 (27.6)	848 (19.7)	
NICU	6040 (72.1)	3389 (79.6)	
Time (24-h clock) of intubation (*n*, %)			<0.001
0800–1559	4139 (49.4)	1495 (35.1)	
1600–2359	2529 (30.2)	1439 (33.8)	
0000–0759	1715 (20.5)	1325.1)	

^a^During the first intubation attempt.

^b^813/3159 (10%) delivery room intubations used premedications, 7111/9434 (75.4%) of NICU intubations used premedications.

### First attempt success and number of attempts

Univariate analysis found attending neonatologist presence was positively associated with first attempt intubation success (OR 1.11, 95% CI 1.04–1.20). However, on multivariate analysis, when controlling for the confounders of prematurity, known history of difficult airway, operator experience, premedication and night-time intubations, attending presence was found to be negatively associated with first attempt intubation success (adjusted OR (aOR) 0.78, 95% CI 0.70–0.86).

Subgroup analysis (Table 2) found attending presence was consistently associated with decreased odds of first attempt success across all levels of operator experience; the strength of the association was greatest for operators with the most experience (aOR 0.67, 95% CI 0.50–0.91). Similarly, stratified analyses by gestational age, neonates with a known history of difficult airways, and the location of intubation also consistently demonstrated a negative association between attending presence and first attempt success.

**Table 2:** Subgroup analyses of the impact of attending neonatologist presence on first attempt intubation success.

Subgroup	Adjusted OR	95% CI
Operator experience (*n*, %)		
0–2 years (3834, 30.3)	0.81	0.69–0.96
3–5 years (6892, 54.5)	0.79	0.68–0.91
6+ years (1926, 15.2)	0.67	0.50–0.91
Gestational age (*n*, %)		
≤28 weeks (6169, 48.8)	0.80	0.69–0.93
>28 to 34 weeks (2919, 23.1)	0.73	0.65–0.83
>34 weeks (3564, 28.2)	0.77	0.62–0.95
Known history of difficult airway (*n*, %)		
Yes (856, 6.8)	0.65	0.51–0.81
No (11,796, 93.2)	0.81	0.73–0.89
Location of intubation (*n*, %)		
Delivery Room (3159, 25.0)	0.66	0.57–0.78
NICU (9434, 74.6)	0.81	0.72–0.91

Multivariate analysis found attending presence was associated with increased odds of intubation requiring three or more attempts compared to successful intubation within two attempts (aOR 1.37, 95% CI 1.21–1.60).

### Tracheal intubation associated events (TIAEs) and severe desaturation

On multivariate analysis, attending presence was associated with increased odds of severe oxygen desaturation (aOR 1.12, 95% CI 1.02–1.24).

Due to the low incidence of many of the reported TIAEs in the study cohort, there was insufficient precision to provide meaningful effect estimates of attending presence on individual TIAEs. The incidence of these data has been presented in Table 3. When these individual events were aggregated into composite outcomes, multivariate analysis found attending presence to be associated with increased odds of both non-severe (aOR 1.29, 95% CI 1.09–1.52) and severe TIAEs (aOR 1.69 95% CI 1.16–2.47).

**Table 3:** Incidence of adverse events.

Adverse event (*n*, %)	Attending neonatologist present (*n* = 8391, 100%)	Attending neonatologist absent (*n* = 4261, 100%)
Severe oxygen desaturation (4595, 36.3)	2975 (35.5)	1620 (38.0)
**Any non-severe TIAE (2143, 24.2)**	**1350 (16.1)**	**793 (18.6)**
Dysrhythmia (inc. bradycardia <60/min) (940, 7.4)	577 (6.9)	363 (8.5)
Mainstem intubation (193, 1.5)	126 (1.5)	67 (1.6)
Esophageal intubation (immediate recognition) (1031, 8.1)	662 (7.9)	369 (8.7)
Vomit (no aspiration) (62, 0.5)	35 (0.4)	27 (0.6)
Epistaxis (17, 0.1)	13 (0.2)	4 (0.1)
Gum/dental trauma (110, 0.9)	70 (0.8)	40 (1.0)
Lip trauma (50, 0.4)	42 (0.5)	8 (0.2)
Pain/agitation requiring additional medications (45, 0.4)	22 (0.3)	23 (0.5)
**Any severe TIAE (398, 3.1)**	**297 (3.5)**	**96 (2.3)**
Death (16, 0.1)	14 (0.2)	2 (0.1)
Cardiac arrest (survived) (46, 0.4)	38 (0.5)	8 (0.2)
Cardiac compression (<1 min) (82, 0.6)	58 (0.7)	24 (0.6)
Hypotension requiring therapy (3, 0.02)	3 (0.04)	0 (0)
Esophageal intubation (delayed recognition) (155, 1.2)	110 (1.3)	45 (1.1)
Pneumothorax/pneumomediastinum (35, 0.3)	26 (0.3)	9 (0.2)
Direct airway injury (45, 0.4)	38 (0.5)	7 (0.2)
Laryngospasm (28, 0.2)	21 (0.3)	7 (0.2)

*TIAE* Tracheal Intubation Adverse Events.

## Discussion

This multicenter retrospective observational study is the first to examine the association between attending neonatologist presence and first attempt intubation success rates and TIAEs in neonates. Attending neonatologist presence during endotracheal intubation was more likely for infants who were larger, older, had a known history of difficult airway, and those intubations that occurred in the delivery room, during the day and with video laryngoscopy. However, these associations were only demonstrated on bivariate analysis without consideration of potential confounders or the strength of these associations, and should be interpreted with caution.

On univariate analysis attending presence was associated with increased likelihood of first attempt intubation success. However, in contrast to our hypothesis, after adjusting for potential confounders attending neonatologist presence was associated with lower first attempt intubation success overall. This association remained across all operator experience and gestational age subgroups. The study also demonstrated an association between attending presence and more episodes of severe desaturation during intubation, non-severe and severe TIAEs (when individual adverse events were aggregated into composite outcomes), as well as a higher number of intubation attempts.

There are several potential explanations for our findings. It is likely there were unmeasured confounders which were not adjusted for, meaning that there was greater illness severity for the group where an attending neonatologist was present, and subsequently lower likelihood of first attempt intubation success as well as greater risk of adverse events. These data suggest that clinical teams may already be risk stratifying to ensure an attending is present at intubations anticipated to be more challenging or risky in their NICU. This is supported by a survey of NEAR4NEOS sites conducted following the present study that found NICUs typically have attendings present for intubations involving infants that are extremely premature, have a known difficult airway, have underlying congenital anomalies conferring a greater risk of clinical instability during intubation (for example congenital heart disease or congenital diaphragmatic hernia) and emergent intubations. Some units reported having guidelines or protocols for attending presence at neonatal intubation. Furthermore, differences in training of operators, and the expertise of the attending in coaching the attempt would be relevant information in future studies.

In addition, with senior presence to escalate subsequent intubation attempts, there may be a greater tolerance for patient instability such as severe oxygen desaturation and multiple intubation attempts, or attendings may terminate intubation attempts earlier leading to lack of success. The psychological effects of an attending on junior and supporting staff are intangible and may increase performance anxiety and operator stress leading to lower first attempt success. The study did not evaluate first attempt success and patient stability when the attending neonatologist was the first operator; previous studies have demonstrated higher rates of first attempt intubation success with increasing operator experience [5, 17]. The reasons for an attending being present are not collected in the registry, nor the timepoint at which the attending neonatologist was present during the intubation. We would recommend that these parameters are collected in the future, in order to further evaluate the impact of attending presence.

Inexperienced operators have been demonstrated to have the lowest rates of first attempt intubation success, as low as 23%, while also having higher rates of TIAEs [4, 16]. TIAEs are relatively common in neonates owing to their airway anatomy and relatively minimal physiological reserve [11, 15]. The results of our study suggest that attending neonatologists were likely appropriately present for anticipated high risk intubations. However, attending neonatologist supervision may not mitigate the risks associated with neonatal intubation.

Attending neonatologist coverage across NICUs varies significantly, as does individual attending coaching skill. Research examining the impacts of increased attending coverage on clinical outcomes is also varied in its findings [26–28]. This study does not delineate the specific aspects of attending presence that contribute to the observed decrease in first attempt success. Future studies are warranted to explore these dynamics in greater detail. It may be that the presence of an attending neonatologist alone is insufficient to influence first attempt intubation success, but instead should facilitate a strategy of matching the operator to the intubation and adjuncts that may increase the chance of success such as video laryngoscopy, premedications and potentially nasal high flow use.

A strength of this study was the use of a DAG for identification of confounders to include as covariates in our statistical modeling. In observational studies exploring casual effects, unnecessarily adjusting for confounders or variables that are not true confounders, can introduce bias leading to erroneous effect estimates [19–21]. The use of a DAG in our study reduces the risk of such bias.

This study has a number of limitations. The observational nature of the study makes it susceptible to unmeasured confounders not identified by the DAG, introducing the risk of bias to causal effect estimates. As data were not collected on changes in practice that may have occurred during the study period, these practice changes could not be accounted for. Attending presence may have altered the completeness of data and led to a selection bias. Years of experience is likely an imprecise measure of neonatal intubation experience, as it does not take into account factors such as variability in terms of numbers of intubations performed, recency of experience, or institutional and regional differences leading to differences in exposure to neonatal intubations. Intubations were excluded if they were missing data for confounding variables, potentially introducing a degree of selection bias.

The modeling of the relationship between attending presence and composite adverse outcomes combined events of heterogeneous significance. This may make the interpretation of the clinical significance of these associations challenging, and thus should be interpreted with caution.

Further research may help to delineate the most effective strategies for supporting intubations where an attending neonatologist is supervising, to optimize coaching, team performance and subsequent procedural success.

## Conclusion

After adjustment for potential confounders and site clustering, attending presence was associated with a lower likelihood of first attempt intubation success, and increased odds of ≥3 intubation attempts and severe desaturation. These results suggest that attending neonatologists may appropriately be more often present in anticipated high-risk intubations, including those with difficult airway, yet alternative strategies to improve intubation success and proficiency of trainees may be more important than attending presence alone. Furthermore, there may be unmeasured confounders that have not been accounted for as a source of bias. Further research is needed to examine operator-related factors that impact intubation success.

## Summary

### What is already known on this topic

- Neonatal endotracheal intubation is an important but high-risk procedure commonly associated with low first attempt success rates adverse events.

### What this study adds

- In this multicentre retrospective cohort study, attending neonatologist presence was associated with lower first attempt intubation success, increased odds of severe oxygen desaturation and composite odds adverse events. Reasons for this may include appropriate anticipation of high-risk intubations and unmeasured confounding biases.

### How this study might affect research, practice or policy

- Mere attending presence may not mitigate risks associated with neonatal intubation unless additional strategies are employed. Future research should refine how best to leverage attending expertise to improve neonatal intubation outcomes.

## Data Availability

Deidentified individual participant data (including data dictionaries) will be made available, in addition to study protocols. The data will be made available upon publication to researchers who provide a methodologically sound proposal for use in achieving the goals of the approved proposal. Data are available upon reasonable request with approval by the NEAR4NEOS Manuscript Oversight Committee. Requests should be directed to buffmanH@chop.edu. Data requestors will need to sign a data access agreement approved by the Children’s Hospital of Philadelphia.
